# Rewriting the narrative: a perspective on youth limb difference, community access and the power of amputee soccer

**DOI:** 10.3389/fspor.2026.1798505

**Published:** 2026-07-31

**Authors:** James Kevin Pierre-Glaude, Adriana Saab, Taylor White, Eric M. Lamberg

**Affiliations:** 1 School of Health Professions, Stony Brook University, Stony Brook, NY, United States; 2 American Amputee Soccer Association, Stony Brook, NY, United States

**Keywords:** adaptive sport, amputation, community program, limb difference, soccer (football)

## Abstract

This perspective addresses an important gap in adaptive sport access for youth with limb loss (LL) and limb difference (LD). Although the potential benefits of sport participation are well documented, accessible adaptive sport opportunities remain limited. This article describes the development and implementation of a youth amputee soccer initiative on Long Island, New York. Guided by a biopsychosocial framework and supported by physical therapists, trained coaches, and adult amputee athlete mentors the program was designed to provide a structured and inclusive environment for skill development, peer interaction and enhanced community connection. Descriptive program data and caregiver reported feedback are presented to illustrate the perceived value of the program. Caregiver responses suggest high satisfaction, positive perceptions of social interaction, and appreciation for the mentorship and inclusive environment. These findings are preliminary yet suggest that intentionally designed, community based adaptive sport programs may contribute to expanding opportunity and supporting participation and belonging for youth with LL and LD.

## Introduction

Today, more than 5.6 million people in the United States live with limb loss (LL) and limb difference (LD) (1). Annually, an estimated 42,000 people are born with LD, and 1.2% of those with LL undergo amputation before the age of 18 (1). Despite these numbers, there are limited organizations nationwide designed to cater to adaptive sports for individuals across the lifespan (2). According to Move United, 4 in 10 individuals with disabilities express a desire to participate in sports, yet 7 in 10 are not aware of organizations that can support them (3, 4). This gap in infrastructure creates a critical barrier to health and social development.

Currently, 3 million children in the United States have a disability, and their activity levels are 4.5 times lower than age-matched peers, contributing to an obesity rate that is 38% higher in this population (4). Unlike their non-disabled peers, children with disabilities cannot easily join standard athletic programs. This lack of access creates a ripple effect where low involvement in community programs often leads to social isolation (5). Furthermore, non-participation affects the entire family unit. Caregivers of children with physical and/or intellectual disabilities are more likely to experience adverse health outcomes themselves (6). Access to adaptive community programs allows caregivers the time to attend to their own social, emotional, and physical needs all while their child fosters their own growth in a supportive environment (7). Research confirms that bridging this gap yields profound results. A study encompassing 163 youth highlighted the importance of adaptive sports for adolescents with chronic diseases or physical disabilities. Participation showed a positive correlation with increased aerobic and anaerobic fitness, agility, muscle strength, and physical activity levels (8). Beyond physical health, adaptive sports significantly improve psychosocial well-being. A study from the *Journal of Strength and Conditioning Research* demonstrated that children who participated in organized adaptive sports reported improved quality of life, increased feelings of athletic competence, greater social acceptance, and higher exercise self-efficacy compared to non-participating peers (5).

Every child has the right to feel included. Yet, due to the limited number of inclusive and adapted sports programs, some individuals must travel significant distances, if they are able, just to have the opportunity to play and participate. Raising awareness of the benefits of adaptive sports is only the first step; it must be followed by the intentional creation of accessible infrastructure. Engaging coaches in the development of this infrastructure is crucial. Amputee soccer, an adaptive sport for individuals with upper or lower LL or LD, with its minimal equipment requirements and team-centric focus, offers an ideal pathway to address these disparities. The following sections outline the sport and the strategic framework for the development of a youth amputee soccer program designed to bridge this gap and bring the sport to the communities that need it most.

### What are adaptive sports?

Adaptive sports are competitive or recreational sports modified to allow individuals with disabilities to participate. Some adaptive sports programs use classifications systems to ensure fair competition (9). Adaptive sports differ from inclusive sports. Inclusive sports allow individuals with and without disabilities to participate together under the same rules, thus bringing equal opportunities (10), diverse perspectives and skills, and promoting social harmony and mutual respect (11).

### Amputee soccer

Amputee soccer (internationally known as amputee futbol) is an adaptive sport governed globally by the World Amputee Football Federation (WAFF). The sport serves individuals with upper or lower LL or LD. The WAFF's mission emphasizes the promotion of psychosocial well-being, specifically targeting the enhancement of self-confidence, self-reliance, and independence through athletic participation (https://amputeefootball.org). Matches are played on a reduced sized field with two 25-minute halves. During a match, each team comprises seven players: six field players, who must have a lower limb impairment and utilize bilateral forearm crutches, and one goalkeeper, who must have an upper limb impairment. The field players are unable to use their residual limb or crutches to manipulate the ball while the goalkeepers are confined to the goal box area and may only use their unimpaired arm for saves. Further modifications include elimination of the offsides rule, the prohibition of slide tackling and the replacement of throw-ins with kick-ins. While the sport is designed to be accessible for all individuals with LL or LD to play at the most elite level requires significant athletic ability in areas such as agility, balance, strength and cardiovascular endurance (12).

In the United States, the American Amputee Soccer Association (AASA) serves as the governing body, advocating for increased awareness and access for individuals with LL and LD (https://www.usampsoccer.org). The AASA operates under a dual mission regarding sport development. First, the association focuses on grassroots and regional growth by promoting the sport across junior and adult demographics, irrespective of gender. Currently sanctioning more than 15 regional teams with additional programs in development, the AASA aims to introduce athletes to the sport, enhance technical proficiency, and foster self-confidence, teamwork and community cohesion. The organization's second mission involves the identification and training of elite athletes for international competition.

### Program development and framework: youth amputee soccer

The primary objective of the youth amputee soccer initiative on Long Island, New York, in coordination with the AASA, is to establish a standardized, reproducible framework that improves regional accessibility while reducing logistical barriers to program development. By developing a replicable operational model, this initiative aims to minimize the logistical barriers associated with establishing new amputee soccer programs. The central goal of these clinics is to cultivate an environment that welcomes athletes and their families, providing an introduction to the sport alongside guided skill development. Additionally, the clinics prioritize social integration, facilitating peer support and camaraderie among children with LD and LL and creating a defined developmental pathway for youth athletes, linking recreational participation to potential pathways toward national and international level competition.

Adaptive sports and recreation are critical for the physical and psychosocial rehabilitation of persons with limb loss; however, success is often dependent on specialized environments that accommodate their unique functional needs (13). Dedicated adaptive programming is essential to mitigate the attrition rates observed when children with LL and LD participate in mainstream athletics. Youth with LL and LD often face distinct biomechanical disadvantages when participating alongside non-disabled peers. In sports that permit prosthetic devices, functional limitations and increased metabolic costs often lead to rapid fatigue (14). This physical disparity prevents athletes from performing at parity with able-bodied peers, potentially diminishing self-esteem; furthermore, the inability to fully participate can inadvertently exacerbate stigma and social exclusion (15, 16). Additionally, mainstream community sports organizations often lack the specialized coaching expertise required to accommodate physical impairments. Consequently, without a specialized coaching staff, children with LD and LL risk exclusion from activity, may have too much attention drawn to them, or may not be provided with the appropriate guidance needed to advance their athletic skills.

**Figure 1:**
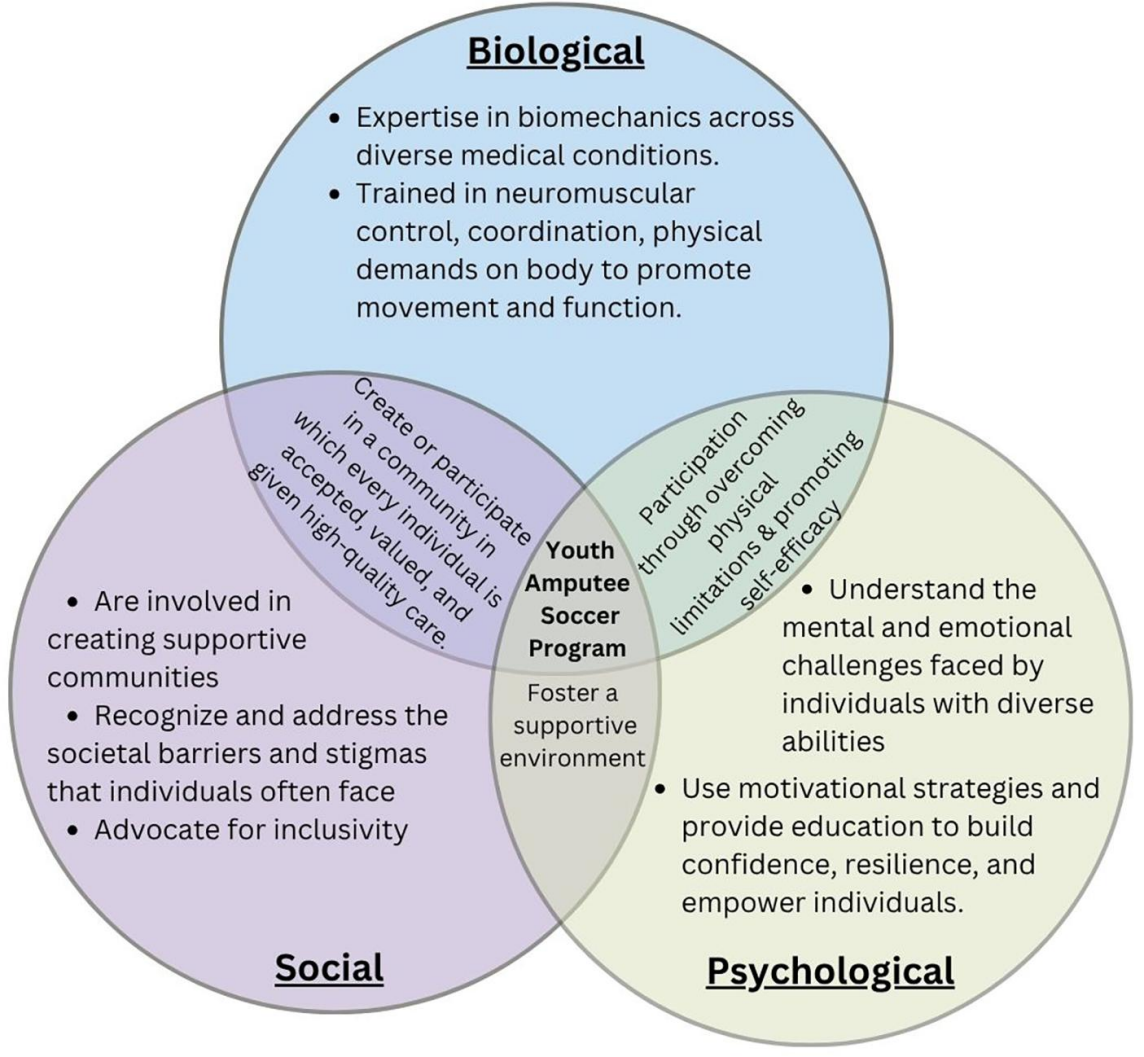
Biopsychosocial model implementation.

The biopsychosocial model, first coined by George Engel in 1977, improved upon the biomedical model by acknowledging that psychological and social factors play a role in health and disease (17). This model was most recently used for understanding athletic training load, fatigue, and musculoskeletal sport injury in university athletes (18, 19). Led by physical therapists with dual expertise in amputee and mainstream soccer, the program utilized a biopsychosocial framework to address athlete development holistically (Figure 1).

While the initiative was originally designed specifically for youth with LL and LD, the application of this therapeutic, evidence-based framework facilitated the expansion of the program. The environment successfully accommodated children with a wider range of physical and cognitive impairments, resulting in a comprehensive, inclusive, and empowering athletic setting for youth of all abilities. To translate this theoretical framework into practice, the program established a standardized structure designed to maximize social inclusion and skill acquisition.

### Soccer clinic structure and implementation

Each 60- to 90-minute session is facilitated by AASA coaches, supported by volunteers and AASA staff, utilizing an inclusive participation model open to youth of all ages. This environment intentionally welcomes children with LL, LD, and varying degrees of gross motor delays, alongside age-appropriate, non-disabled peers. Designed to accommodate all levels of soccer knowledge, the clinics encourage peer modeling and allow novice players to observe and learn from those with more experience. To facilitate this development, sessions follow a consistent framework consisting of a dynamic warm-up, structured skill-building, and inclusive scrimmage play. Warm-ups emphasize balance, coordination, dynamic movement, and safe assistive device use, while the skill development phase targets foundational mechanics through fun, game-based activities. During these drills, coaches implement motor learning principles to guide program progression, utilizing task breakdown (part-to-whole practice), structured progression from closed to open environments, and multimodal (verbal, visual, and tactile) cueing. Furthermore, youth clinics are intentionally scheduled to coincide with adult AASA team practices to foster an intergenerational approach. This allows the sessions to culminate in an integrated youth and adult scrimmage, where a buddy system is utilized to provide mentorship, concurrent feedback, and real-time skill application. Each session ends with a “unity huddle,” reinforcing the core values of acceptance, teamwork, and community. Additional details regarding session structure, coaching strategies, program progression, and evaluation procedures are provided in supplementary file 1.

## Program evaluation: challenges and successes

### Barriers to implementation

The initial outreach phase for recruitment presented the most significant structural challenge. A comprehensive contact database of over 150 organizations was compiled, targeting local medical professionals, community soccer organizations, prosthetists, amputee support groups, departments of recreation for local towns and rehabilitation clinics and facilities. Despite a multimodal recruitment strategy utilizing email, direct calls, in-person site visits, and social media dissemination, the response rate was notably low, with several entities requesting removal from communication lists.

This resistance highlights broader systemic barriers facing the LL and LD community, including a pervasive lack of awareness regarding physical capabilities, social exclusion, and limited representation in mainstream media. Consequently, the inaugural clinic hosted only a single attendee. However, this initial low turnout established a critical baseline. Rather than failure, it represented the necessary first step in establishing a presence, which catalyzed increases in participation by subsequent sessions. Overall, between the dates of March 2024 and November 2025 this program held 14 clinics and served 39 unique participants (10 with LL or LD: 1 proximal femoral focal deficiency, 1 hemipelvectomy, 1 rotationplasty, 2 transfemoral amputation, 3 transtibial amputation and 2 below elbow amputation). The frequency of attendance for all participants ranged from 1 to 7 sessions.

### Outcomes and impact

Financial assistance was secured through the Suffolk County, New York Youth Team Sports Grant, which supported program development including space rental and equipment needs. A distinctive feature of this program was its integration with the regional adult amputee soccer team, which created opportunities for youth participants and their families to interact with adults ho ave lived experience with LL and LD.

To capture participant feedback, an anonymous custom questionnaire was developed. Categorical and qualitative questions were designed to allow for the collection of post-clinic feedback. Although the questionnaire was reviewed by three of the study authors and one independent external reviewer to improve clarity and ease of comprehension, no formal validation study was conducted. Feedback was integrated into the final version of the form that was used (supplementary file 2). Categorical data was summed and is presented as an aggregate. Open-ended feedback did not undergo formal qualitative evaluation. Instead, responses were reviewed, and representative sample statements were extracted to provide illustrative examples into the participant experience.

Data was obtained from 11 parents and caregivers of the participants, with responses indicating high parent and caregiver satisfaction (supplementary file 3). All respondents (100%) rated their overall clinic experience and pre-clinic communication as a 5 out of 5 (5 represents excellent). Furthermore, 100% of respondents agreed the clinics were well-organized (with 91% rating organization a 5/5) and reported that their children had “a lot of fun.” The specialized coaching staff was well received with 100% of parents rated the staff as “knowledgeable,” and 91% additionally described them as “approachable” and “good with kids.” In terms of athletic development, 73% of parents reported their child learned “a lot” of new soccer skills, while an additional 18% noted learning “a little,” and 9% stated “not much.”

Beyond athletic skill, the responses from parent and caregivers also highlighted perceived psychosocial benefits associated with program participation. All parents (100%) identified “social interaction” as a primary benefit of attendance, with 73% additionally noting “increased confidence” and “skill improvement.” When asked about peer interaction, 91% of parents reported their child was either very satisfied (55%) or satisfied (36%) playing alongside peers. Because these observations were based on parent and caregiver report using a non-validated questionnaire, they should be interpreted as perceptions rather than evidence of measured psychosocial or functional change.

Open-ended comments offered additional insight. Parents emphasized that even if a child was not inherently drawn to soccer, the camaraderie among peers with similar lived experiences was a primary motivator for continued attendance. Because no formal qualitative methodology (e.g., coding, thematic analysis, or content analysis) was performed, the comments below in response to the prompt, “What did participation in these clinics mean to you (as the parent) and to your child?” are included solely as illustrative examples of parent and caregiver perspectives and are not presented as systematically analyzed qualitative findings.

“(Child's name) is now interested in playing soccer so this helps build her confidence.. It made her feel that she is able to do it.”

“My son has been extremely upset watching his identical twin brother play sports. It's all he wants to do.”

“[He] learned to love soccer by watching college athletes play it.. after his first amputee soccer clinic, he verbalized ‘amputee soccer is my best type of soccer’ and saw a version of soccer he could compete and excel in.”

“belonging, community, confidence”

“It's very great for my son. Increases his confidence!”

“Socialization.. inclusion.. acceptance and fun”

“Watching my son learn the game and also watching professionals was amazing. We all enjoy and appreciate this community so much.”

When asked about barriers to attendance, parents and caregivers the reported logistical challenges, such as scheduling conflicts (*n* = 6), clinic distance (*n* = 3), and transportation (*n* = 1). Nevertheless, the program's overall outcomes suggest perceived success within this context. These outcomes specifically address two persistent systemic barriers to adaptive sport participation: the ‘lack of accessible and inclusive opportunities’ and a limited number of trained volunteers. The deployment of a highly knowledgeable coaching staff and the provision of a specialized, inclusive environment suggest this program model may help address perceived barriers within this local context.

## Discussion

The parent and caregiver feedback obtained from this program suggests that families valued the opportunity for structured adaptive sport participation, peer connection, and engagement with adult amputee athlete mentors. These observations are consistent with broader literature describing the potential biopsychosocial benefits of the sport within the American Amputee Soccer Association community (20) but in the present work they should be interpreted as descriptive and exploratory.

A distinctive feature of this initiative is the integration of youth clinics with adult amputee soccer team activity. This intergenerational structure may provide a particularly meaningful source of role modeling, mentorship, and community connection for youth with LL and LD and for their families. In the caregiver comments collected during this program, families frequently emphasized belonging, confidence, and the importance of seeing older athletes with similar lived experiences. Although these perspectives cannot be interpreted as formally measured psychosocial outcomes, they highlight a potentially important feature of this program design that warrants further study.

The program was also developed within a biopsychosocial framework and led by clinicians and coaches familiar with both amputee rehabilitation and soccer. In practice, this framework supported attention to physical skill development, social participation, and inclusive coaching strategies. It may also offer a useful model for organizing future adaptive sport programming. At the same time, the information gathered from this program does not establish clinical rehabilitation benefit, psychosocial improvement, or long-term participation effects.

A unique benefit of amputee soccer is the opportunity to practice non-prosthetic mobility. While rehabilitation for those with LL and LD often prioritizes prosthetic use, the reality for pediatric patients is that reliance on a device can be limiting. Frequent growth spurts necessitate socket adjustments and replacements, often leaving children without a functioning prosthesis for extended periods. Through soccer, participants were provided the opportunity to develop the skill and confidence to maintain mobility through use of crutches independent of their prosthetic device.

Consistent with research indicating that children with physical disabilities report higher social acceptance when participating in sports, this program creates a community where ability is emphasized (21). Caregiver perspectives suggest that the clinics offered an environment in which their children and the families felt was inclusive and supportive as compared to experiences in mainstream sport settings.

### Limitations

This manuscript should be interpreted in light of several limitationss. Generalizability is limited by a small sample size (11) and reliance on parent and caregiver reported outcomes collected at a single time point using a custom, non-validated questionnaire, which may introduce response and selection bias. Outcomes were not obtained directly from participating children, limiting insight into the athletes’ firsthand experiences. Additionally, open ended feedback was not analyzed using formal qualitative methods, restricting the depth and rigor of thematic interpretation. As such, the reported findings are best understood as preliminary and descriptive indicators of program impact rather than definitive evidence of efficacy. Finally, the program was implemented within a single geographic region and benefited from factors such as access to specialized clinicians and an established adult amputee soccer team.

### Implications and future directions

This initiative illustrates how a community based adaptive sport program for youth with LL and LD can be organized around a biopsychosocial framework that is led by clinicians with sport-specific expertise and paired with intergenerational mentorship. Within this program families viewed the clinics positively and reported perceived social and developmental value. As such, the program may serve as a useful example for those interested in building similar opportunities.. Future work should focus on strengthening the foundation of this program model through longitudinal study designs, incorporation of validated physical and psychosocial outcome measures, and direct inclusion of child reported perspectives. Additionally, further exploration of program replication across diverse geographic and resource settings, including coach education and partnerships with community organizations will be essential to expanding access.

## Conclusion

The youth amputee soccer initiative on Long Island provides a descriptive, early-stage example of how community based adaptive sport programming can be developed for youth with LL and LD.. Caregiver feedback suggests that families valued the programs structure, mentorship opportunities and social environment. This perspective is intended to highlight the practical lessons from program development to inform replication in other areas such that access to adapted sport is more readily available.

## Data Availability

The raw data supporting the conclusions of this article will be made available by the authors, without undue reservation.
