# Supplementary material for: Rewriting the narrative: a perspective on youth limb difference, community access and the power of amputee soccer
Source: Front Sports Act Living. 2026 Jul 31;8:1798505. doi: 10.3389/fspor.2026.1798505 (PMC13473271; doi:10.3389/fspor.2026.1798505)
Supplement: Supplementary file 1 [file Supplementaryfile2.pdf]

Youth Amputee Soccer Clinics Feedback Survey

Thank you for allowing your child to participate in the soccer clinics hosted by the American Amputee Soccer Association! Your feedback is invaluable in helping us improve our offerings and the experiences for participating families. All responses are anonymous.

Reach out to [redacted] or [redacted] with any questions. Thank you!

* Indicates required question

1. How would you rate your overall experience at the clinics? *

Mark only one oval.

	1	2	3	4	5	
Poo	◯	◯	◯	◯	◯	Excellent

2. How would you rate the communication before and during the clinic?

Mark only one oval.

	1	2	3	4	5	
Poo	◯	◯	◯	◯	◯	Excellent

3. How well organized was the clinic?

Mark only one oval.

	1	2	3	4	5	
Poo	◯	◯	◯	◯	◯	Excellent

4. How would you rate the coaching staff (select all that apply)

Check all that apply.

- [ ] Knowledgeable
- [ ] Approachable
- [ ] Good with kids
- [ ] Helpful
- [ ] Other: ____________________

5. How do you feel your child(ren) benefitted from the clinic? (select all that apply)

Check all that apply.

- [ ] Skill improvement
- [ ] Increased confidence
- [ ] Social interaction
- [ ] Other: ____________________

6. Did your child have fun at the clinic?

Mark only one oval.

- A lot of fun.
- Some fun.
- Not much fun.

7. Did your child learn new soccer skills?

Mark only one oval.

- Yes, a lot
- A little
- Not much

8. Did you and your child enjoy the games and drills?

Mark only one oval.

- Yes, they were fun!
- They were okay.
- We didn't like them

9. What did participation in these clinics mean to you (as the parent) and to your child? *

10. What was your child's favorite part of the clinic? *

11. What was your child's least favorite part of the clinic? *

12. How did your child feel playing soccer with peers? *

Mark only one oval.

- Very Satisfied
- Satisfied
- Neutral
- Unsatisfied
- Very Unsatisfied

13. Were there any reasons preventing you from attending a clinic? *

Mark only one oval.

- Transportation
- Distance of clinic setting
- Scheduling conflicts
- Other:

14. Is there anything you wish to change about the clinics? If yes, please write in the "other" section below

Mark only one oval.

- No
- Other:

15. Do you have any comments or concerns you would like to state? If yes, please write in the "other" section below

Mark only one oval.

- No
- Other:

This content is neither created nor endorsed by Google.

Google Forms
