# Supplementary material for: Rewriting the narrative: a perspective on youth limb difference, community access and the power of amputee soccer
Source: Front Sports Act Living. 2026 Jul 31;8:1798505. doi: 10.3389/fspor.2026.1798505 (PMC13473271; doi:10.3389/fspor.2026.1798505)
Supplement: Supplementary file 2 [file Supplementaryfile1.docx]

**Supplemental 1**

**Session structure**

Each session is 60-90 minutes in duration and follows a consistent structure.

1. Warm up and dynamic mobility
   1. At the beginning of each session, players are led through a warm up emphasizing balance, coordination, safe crutch/assistive device/prosthetic use, and flexibility. Activities include dynamic stretching exercises, running drills (including changes in direction), and partner based movement exercises adapted for each player’s physical abilities. During the warm up, coaches are stationed throughout to provide modifications, feedback, and positive reinforcements (high-5s, vocal affirmations).
2. Skill development
   1. Players participate in individual or team-based structured skill-building drills targeting foundational soccer skills (dribbling, passing, shooting, spatial awareness, game rules). Skill development drills are conducted through a games-based approach, adapted from familiar youth activities (capture the flag, object retrieval games, freeze tag, monkey in the middle, relay races, obstacle courses, target accuracy). Facilitating “fun” structured drills gives the ability to promote engagement, motivation, and repetition. All drills are modified during implementation to accommodate varying abilities and accessibility.
3. Scrimmage
   1. Clinic sessions conclude with full group scrimmage play integrating both youth and adult players. Teams are intentionally determined with adult players paired with a youth participant (buddy system) to facilitate a mentoring dynamic. Adult players provide concurrent feedback while being a role model and aiding in youth development and confidence. This dynamic reinforces skill development, creates an empowering environment, and strengthens the sense of community.
4. Unity Huddle
   1. Sessions end with a "unity huddle," reinforcing the core values of acceptance, teamwork, and community among youth players, adult players, and coaches

**Coaching strategies / program progression**

- The fundamentals of motor learning
  - Practice styles can be arranged in a variety of ways. Whole practice describes practicing a skill in its entirety, such as dribbling and shooting a ball. Part practice refers to focusing on one aspect of a multi-step skill such as isolating shooting on goal. Blocked practice involves repeated exposure to one aspect of the skill such as repeated practice shooting on goal. By isolating each section of a skill, a player is able to accomplish mastery; and reintroduce all aspects of the skill to reinforce their understanding and performance.
  - Environment: Providing a controlled, closed environment allows for improved learning. By practicing and learning individually in a closed environment, such as a single goal within a small section of field; this allows each child to focus on the skill being presented without distraction encountered in open environments (full field or 2+players). Once a skill has been sufficiently learned, it can be introduced into an open environment, through the addition of more peers, or applied in a scrimmage setting.
  - Cueing: Tactile, visual and verbal cueing are most commonly used in order to facilitate gross motor learning. Tactile cueing refers to hands-on guidance such as tapping on a child’s lower extremity to encourage a correct step/crutch and kick sequence. Verbal cueing describes implementing words in order to facilitate performance, such as “fast, slow, left, right, big, little, etc.” These verbal cues help to enhance the quality of performance. Visual cueing is the use of markers or other objects that serve as reminders or assistance for the child while performing a new or learned skill. This can include colored spot markers, shapes, cones, stickers, etc that an individual associates with the skill. For example, cones can strategically be placed to facilitate the path of motion or target.
  - Modeling: the use of visual demonstration and peer modeling are integral parts of motor learning. For a new skill, providing a visual demonstration allows the expectation of the skill to be clear and translate more effectively than verbal cueing alone. The use of peer modeling is a tool that can provide additional visual feedback to a learner. Being able to observe an age-matched or otherwise ability-appropriate peer performing a skill can enhance comprehension when compared to a child watching an adult perform an unknown skill.
- Motivation
  - Maintaining high morale creates a productive learning environment. Positive reinforcement from instructors as well as peers provides feedback that can help to fuel confidence. Motivating factors can also come from the use of rewards such as stickers, preferred activities, dancing, prizes, etc.
- Gross motor building blocks
  - Working on fundamental gross motor skills through adaptive activities is a fun way for children to progress these skills. For example, asking a child to raise one foot, place it on a ball and hold that position is targeting balance, lower extremity strength, upper extremity strength if use of crutches, endurance, proprioception, trunk stability, and reactive balance strategies.
  - Adaptive balance strategies involve the nervous system learning to react to previously encountered conditions or activities such as walking across a slippery surface, or walking across a balance beam. The more an individual is exposed to the task, the better their body learns to adapt to known conditions. For example, asking a child to dribble a soccer ball and shoot into a goal involves forward momentum and intentional action to balance, stop, and kick. A child practicing this skill allows them to improve their adaptive balance strategies, understanding how their body needs to adjust in order to maintain their balance to aid in shooting.
  - Reactive balance strategies describe the way our body adapts to unknown conditions. For example, practicing a drill in which a child is running or dribbling a soccer ball; and is suddenly prompted to stop or change direction. This activity allows the child’s nervous and musculoskeletal system to learn to work together to maintain their balance during sudden changes in environment.
  - Other gross motor skills that are addressed through multidisciplinary drills include bilateral coordination (our body’s ability to use both our left and right sides in tandem), hand-eye and hand-foot coordination (our ability to use visual input to guide hand or foot movements), spatial awareness(the brain's ability to understand the body's position in relation to surrounding objects, people, and the environment) and proprioception (internal, unconscious sense of your body’s position and limb movements).

**The importance of intergenerational integration, modeling and multidisciplinary involvement**

- As mentioned above, sessions are conducted alongside adult team practices. This allows opportunity to observe the team playing, and allows adult players to join into youth sessions to provide demonstration and modeling.
- Clinics are facilitated by physical therapists, physical therapy students and pre-physical therapy students. The presence of trained and passionate individuals provides skilled hands-on support and general learning support. Thus allowing greater occurrence of 1:1 assistance for individuals who would benefit.
- Age-appropriate, non-disabled peers are welcome at clinics to facilitate an inclusive environment.

**Program progression**

- With the previously mentioned support and learning tools, children are able to attain new skills and make progress in previously learned skills. As children progress their skills, their reliance on 1:1 support or other cueing styles gradually decreases. Their learning is still supported through the introduction of higher level gross motor skills, challenges to improve their endurance, and opportunity to synthesize all their learned skills in scrimmage.

**Evaluation procedures**

- Attendance and Retention: Attendance was secured at each session to assess consistency of participation
- Coaching Observations: At the conclusion of each session, coaches endured a discussion of each athlete’s engagement, skill development, and social interaction.
- Feedback: To capture participant post-clinic feedback, a custom questionnaire was developed to assess categorical and qualitative data (Appendix 1)
