# Supplementary material for: Rewriting the narrative: a perspective on youth limb difference, community access and the power of amputee soccer
Source: Front Sports Act Living. 2026 Jul 31;8:1798505. doi: 10.3389/fspor.2026.1798505 (PMC13473271; doi:10.3389/fspor.2026.1798505)
Supplement: Supplementary file 3 [file Supplementaryfile3.docx]

**Supplemental 3: Feedback Outcome**

**Categorical Outcomes**

| **ID** | **Experience (out of 5)** | **Communication (out of 5)** | **Organization (out of 5)** | **Coaching Staff Rating** | **Perceived Benefits** | **Had Fun?** | **Learned New Skills?** | **Enjoyed Games/Drills?** |
| --- | --- | --- | --- | --- | --- | --- | --- | --- |
| 1 | 5 | 5 | 5 | Knowledgeable | Skill improvement, Increased confidence, Social interaction | A lot of fun. | Yes, a lot | Yes, they were fun! |
| 2 | 5 | 5 | 4 | Knowledgeable, Approachable, Good with kids | Skill improvement, Social interaction, Validation of amputee soccer as his best type of soccer | A lot of fun. | Yes, a lot | Yes, they were fun! |
| 3 | 5 | 5 | 5 | Knowledgeable, Approachable, Good with kids, Helpful | Skill improvement, Increased confidence, Social interaction | A lot of fun. | A little | Yes, they were fun! |
| 4 | 5 | 5 | 5 | Knowledgeable, Approachable, Good with kids, Helpful | Skill improvement, Increased confidence, Social interaction | A lot of fun. | Yes, a lot | Yes, they were fun! |
| 5 | 5 | 5 | 5 | Knowledgeable, Approachable, Good with kids, Helpful | Increased confidence, Social interaction | A lot of fun. | Yes, a lot | They were okay. |
| 6 | 5 | 5 | 5 | Knowledgeable, Approachable, Good with kids, Helpful | Social interaction | A lot of fun. | Yes, a lot | Yes, they were fun! |
| 7 | 5 | 5 | 5 | Knowledgeable, Approachable, Good with kids, Helpful | Skill improvement, Increased confidence, Social interaction | A lot of fun. | Yes, a lot | Yes, they were fun! |
| 8 | 5 | 5 | 5 | Knowledgeable, Approachable, Good with kids, Helpful | Skill improvement, Increased confidence, Social interaction | A lot of fun. | A little | They were okay. |
| 9 | 5 | 5 | 5 | Knowledgeable, Approachable, Good with kids, Helpful | Skill improvement, Increased confidence, Social interaction | A lot of fun. | Yes, a lot | Yes, they were fun! |
| 10 | 5 | 5 | 5 | Knowledgeable, Approachable, Good with kids, Helpful | Social interaction | A lot of fun. | Not much | Yes, they were fun! |
| 11 | 5 | 5 | 5 | Knowledgeable, Approachable, Good with kids, Helpful, Kind | Skill improvement, Increased confidence, Social interaction | A lot of fun. | Yes, a lot | Yes, they were fun! |

**Qualitative Feedback and Programmatic Barriers**

| **ID** | **Meaning of Participation** | **Favorite Part** | **Least Favorite Part** | **Peer Play Satisfaction** | **Barriers to Attendance** | **Suggested Changes** |
| --- | --- | --- | --- | --- | --- | --- |
| 1 | My son has been extremely upset watching his identical twin brother play sports it’s all he wants to do | Playing with kids like him | None | Very Satisfied | Scheduling conflicts | No |
| 2 | XX learned to love soccer by watching college athletes play it. He has tried to emulate them, but after his first amputee soccer clinic, he verbalized “amputee soccer is my best type of soccer” and saw a version of soccer he could compete and excel in. | Game | N/A | Very Satisfied | Distance of clinic setting | I hope to practice more, once every two weeks. |
| 3 | It’s very great for my son. Increases his confidence! | Competing | Drills | Very Satisfied | Scheduling conflicts | Maybe teach the rules of the game |
| 4 | A lot. XX is now interested in playing soccer so this helps build her confidence so she can try to play next Spring. It made her feel that she is able to do it. | Playing a game | Drills LOL | Satisfied | Scheduling conflicts | No |
| 5 | belonging, community, confidence | game play at end with the adult team | Play game with adults | Neutral | Scheduling conflicts | Hope to keep them for Sundays |
| 6 | Excellent! | Running around | The drill voice during a session | Satisfied | Scheduling conflicts | No |
| 7 | Socialization... inclusion... acceptance and fun | Game | N/A | Very Satisfied | Distance of clinic setting | No |
| 8 | Watching my son learn the game and also watching professional was amazing. We all enjoy and appreciate this community so much. | Kicking goals and doing his celebration dance or spider man pose | That we have to go home and would like more time. | Satisfied | Transportation | No |
| 9 | It means a lot to have somewhere to bring my son with a disability. He asked often to play and to have a place that welcomed him no matter the skill level or what he can do and just let him kick the ball around means a lot! | Red light green light. He always talks about how much fun it was kicking the ball and doing that game! | Nothing he loved it | Very Satisfied | Scheduling conflicts | No |
| 10 | He really liked seeing the adult team play and it was great to meet other children and families who looked like him. | The soccer game at the end | Warm ups | Satisfied | Distance of clinic setting | No |
| 11 | Their self confidence and skills are improving as well as social skills. | The games and the staff | Not sure | Very Satisfied | Saturday is a better day | No |
